# Phenomena of Respiration

**Published:** 1845-12

**Authors:** 


*Phenomena of Respiration.* By M. Vierordt.—M. Vierordt has been making a number of experiments on respiration, and especially on the quantity of carbonic acid exhaled according to the frequency of the respiratory movements. He found that the volume of respired air contained less carbonic acid in proportion as the respirations were more frequent. But the quantity of carbonic acid expired per minute was the same whether the respirations were quick or slow. This law was verified whatever was the quantity of carbonic acid formed per minute,—a quantity, however, which in M. Vierordt at different times varied from 174 to 470 cubic centimetres per minute.—*Ibid. Comp. Ren.*

